# Commissions in Lunacy

**Published:** 1849-07-01

**Authors:** 


### Medical Jurisprudence in relation to Insanity.

### COMMISSIONS IN LUNACY.

(*Exclusively reported for this Journal.*)

Court of Chancery, Westminster, April 25.

IN RE ANSTIE (LUNACY).

Two petitions were presented in the name of William Bailey, of Pentonville, described as secretary of "The Alleged Lunatics' Friend Society," praying that a commission, in the nature of a writ *de lunatico inquirendo*, might issue, to inquire into the lunacy of Peter Sharp Anstie and Henry Anstie. The petition stated, that the two Ansties were residing in the house licensed for the reception of lunatics at Fishponds, near Bristol, and that they were, and had been for many years last past, lunatics, and unfit to manage themselves or their affairs; that there was good reason to believe that the lunatics had been totally neglected by their relatives, and that their property was not duly protected; that each of the lunatics was entitled to an annuity of 350*l.*, devised by the will of their father for the express purpose of being applied for their board and lodging, and all proper care and attendance of their persons, so as to administer to them as many comforts as their state and circumstances would admit; yet that the sum of 110*l.* each per annum had only been applied for some years past for the support of the two lunatics at Fishponds; and that they had been kept in a department appropriated to and living with the noisy, dirty, idiotic, and lowest order of patients, and in rooms of the most cheerless and uncomfortable description, without those comforts which their state and circumstances admitted of, and to the great detriment both of their bodies and minds; and it therefore prayed the issuing of a commission, as before stated. Two other petitions were also presented on behalf of the brothers of the lunatics, also seeking for a commission and the conduct of it. The whole petitions were heard together.

Mr. Malins (Mr. Joliffe with him), for Mr. Bailey, went into the history of the family, whereby it appeared that the father of the two unfortunate lunatics placed them in the asylum at Fishponds, the one forty years and the other thirty years back, and by his will left them an annuity of 350*l.* each for their support. Upon the death of the father, one of his sons,

Benjamin Anstie, looked after the lunatics up to the time of his death, in 1843. Since his death, however, the trustees, through the instrumentality of the next of kin of the lunatics, had cut down the allowance for their support to the paltry sum of 110*l.* each, and, consequently, their treatment had been extremely hard. The affidavit of Mr. Bailey went to the effect, that the lunatics had been entirely neglected by their relatives; that they were confined in the worst department of the asylum with the lowest order of patients; and that they were never taken out for exercise, and kept in a most pitiable state; that they were lodged at night in miserable rooms, and by day in low, dark, common rooms, with dismal paved yards, surrounded by buildings two stories high on three sides, and on the fourth by a high wall. Affidavits of Dr. O'Bryen and Dr. Davis were then read as to the nature of the rooms occupied by the lunatics; and from them it appeared that they had been confined in very small, low rooms, with only one window, and opening into the yard surrounded with buildings; that the floor was a stone one, without any carpet, and that the bed had no furniture, and without any other accommodation whatever in the room. The affidavits then went on to state, that the lunatics were in that part of the establishment allotted to third-class inmates, as regarded social position, payment, and comforts; and that their day-room was shared by persons in humble life; and concluded by a recommendation, that the lunatics should be placed in a more cheerful situation, where they might have the benefit of air and exercise, and an opportunity of having their minds diverted by agreeable scenes and objects, as a means of improving their condition, and adding to their comforts and happiness, and prolonging their lives. Other affidavits were then read, to show that the relatives of the lunatics had entirely omitted of late years to visit them, or to pay any attention to the mode in which they were treated, and that the rooms they were confined in at Fishponds were no better than dismal cells. Some, also, spoke to acts of great violence having been exercised towards the lunatics, and also to the filthy state they were allowed to remain in, solely from the want of proper attendance and regular change of linen. The accumulations from the small proportion of the lunatics' incomes (220*l.* out of 700*l.* a year) being applied by the trustees, amounted to upwards of 30,000*l.*, and it appeared that a part of them, amounting to nearly 9000*l.*, had been divided amongst the brothers and sisters of the lunatics, in preference to the increasing of the comforts of the lunatics themselves. Under such circumstances, it was urged, there being no doubt whatever that the unfortunate gentlemen were confirmed lunatics, the commission as prayed ought to be granted to Mr. Bailey, with a view of having the affairs of the lunatics placed in different hands, and that the petitions of the brothers ought to be dismissed with costs, as they had proved themselves unworthy of having anything further to do with their afflicted relatives.

Mr. Rolt and Mr. Follett, for the next of kin of the lunatics, defended the course of treatment pursued towards the lunatics as having been the best for them under the deplorable circumstances of their cases. First of all, the character of Mr. Bompas, the superintendent of the Fishponds Asylum, was of the highest description, as evinced by the testimony of numerous gentlemen who were well acquainted with him, and the lunatics themselves had been placed in his establishment by their own father. Then with regard to the expenditure for the support of the lunatics, the will of the father plainly contemplated that the whole amount of the annuities would not be required for that purpose; for it spoke of the accumulations being for the lunatics themselves, if they should ever be fortunately restored to reason, and if that should never be the case, then they were to go generally amongst the remainder of his family; all that had, therefore, been done by the division of the 8400*l.* amounted only to an anticipation in favour of the reversioners, as there was no chance whatever that either of the Ansties would ever recover. The next point was the treatment of the lunatics, which had been characterized as cruel in the extreme, both on account of certain violence which had been at times used, and also on account of the stone floors in the rooms, and the absence of covered furniture. The real truth was, that one of the results of the unfortunate malady with which these gentlemen were afflicted was a weakness of body, which rendered it utterly impossible to have anything decent about them. No furniture could have been kept a day without being spoiled with the filth; and medical men themselves had stated, that the best remedy had been used in the present instance—namely, a room with a stone floor properly heated by stoves. With respect to the charge that the lunatics had been neglected by their relatives, there was no truth in it; for they had only ceased to visit their afflicted brothers for the last few years, since they had fallen into such a hopeless state that they could not even recollect their visitors. Looking at the whole circumstances of the case, therefore, there was nothing in it to take it out of the ordinary course, which was, to grant the conduct of the commission to the next of kin of the lunatics.

The Lord Chancellor, without calling for a reply, said the only point for his consideration was who should have the conduct of the commission, as without doubt a commission must issue. Mr. Bailey, a stranger, had stepped in and claimed the conduct of the commission, and therefore it was his place, not only to make out a case for the interference of the court, but also that the next of kin ought not to be intrusted with the inquiry. The fact that Mr. Bailey belonged to a society for the protection of alleged lunatics, did not diminish his claim to be intrusted with the commission, for the society had been instituted for the most benevolent purpose; and he (the lord-chancellor) was sorry to say that, notwithstanding the vigilance of the court, such a society was required. Mr. Bailey presented his petition in the month of February, and the petition of the next of kin was not presented until March; the presumption, therefore, was, that they would never have presented theirs at all, had it not been for Mr. Bailey's coming to the court. Why, if they wished to place their two brothers under the protection of the court, had they not applied years ago? Not only did they not do that, but, when Mr. Bailey had presented his petition, they immediately prepared one having in view the defeat of that of Mr. Bailey. This, he was of opinion, did not afford any ground for depriving Mr. Bailey of the commission, which must without doubt be issued. His lordship then alluded to the facts of the case; and said that the question of extra expenditure was often put in competition with a harsher mode of treatment of the lunatic; for, if the property of the lunatic were not sufficient to meet the expense of extra attendance, &c., the lunatic was obliged to be subjected to harsher treatment as a violent pauper. This, however, could only be excused where the property of the lunatic would not meet the expense of additional comforts. In the present case, the lunacy had been productive of certain habits in the unfortunate gentlemen which could only be kept in check by extra attendance, constant change of linen, &c., or more restraint. Could any one, however, suppose that, looking at their means, these gentlemen ought to be kept in a room with a stone floor, without furniture? He (the lord-chancellor) was of opinion that a considerable addition in the expenditure, for the purpose of increasing the comforts of the lunatics, would be not only justifiable, but really necessary. The question, however, of the mode of treatment, was not now the one before him, but whether the gentleman (Mr. Bailey), who could have only a benevolent object in view, should be deprived of the conduct of the inquiry which he had set in motion. He (the lord-chancellor) thought he ought not to be so deprived. He could not, however, pass over in silence the conduct of the relations of the lunatics, which was altogether unjustifiable, and might, if not commented upon, afford an example for a hundred other cases. That relations were at liberty to cut down the annual expenditure of a lunatic for the purpose of making a fund, to be divided amongst themselves, was a doctrine of a most dangerous description, and would tend, if allowed to be true, to every possible injustice. Without, therefore, entering into the question, how far the comforts of the lunatics ought to be increased, he should decide that a commission of lunacy must issue, and that the conduct of it should be intrusted to Mr. Bailey, and that the petitions of the brothers of the lunatics be dismissed with costs.

---

*Commission de Lunatico Inquirendo, held before* F. BARLOW, ESQ., *one of Her Majesty's Masters in Lunacy, and a Special Jury, as to the state of mind of* CATHERINE BATTAGLIA, *at the Black Horse, Enfield Highway, in the County of Middlesex, on Thursday, the* 16*th of March.*

MR. LLOYD, counsel, and MR. W. FISHER, solicitor, appeared in support of the Commission; Mr. LUCINA, firm of Stevens, Lancaster, and Co., attended to oppose the Commission.

Mr. Commissioner BARLOW opened the case in the manner following:—Gentlemen of the Jury, you are called upon to inquire into the state of mind of Catherine Battaglia. I will state to you as simply and as shortly as I can, the duty you have got to perform, and why it is you are called upon to make this inquiry. You are called upon to make an inquiry of this kind, as in the terms of the Commission, to ascertain the state of this lady's mind; in which there are three questions, namely—Is she a lunatic, an idiot, or a person of unsound mind? It is stated in the Commission, that she is at present residing in Green-street, Enfield Highway; and it is alleged that she is not sufficient for the government of herself, her lands, and tenements; and if this should be found to be the case, the Commission directs you also to inquire into the state of her property; but with that, however, you will have nothing to do in this court, because that matter will be properly investigated before another tribunal. The inquiry before you to-day, therefore, will be limited to that part of the Commission, which directs you to inquire into her state of mind. The first question before you, will be to say, whether you think she is an *idiot*, or a *lunatic*, or a person of *unsound mind.* Now I should recommend you to adopt the simplest term you can imagine, providing you think she is not sufficient for the government of herself and property—namely, a person of *unsound mind.* Each of these terms has a different meaning in law. A person may have been a lunatic, and perhaps is not now a person of unsound mind. That party may, or may not, have recovered his or her intellectual faculties entirely, or at different periods only. The chief question before you to-day, however, is whether you think this person an *idiot*, or a person of *unsound mind*, and the simplest expression will be the best to use. But, looking at that question, you are not to be satisfied with any little eccentricities that the party may have, and reduce them to a state of lunacy, but you are to be satisfied that she is incompetent to take care of herself; or, that at the present time, she is a person of sound mind, and competent to to take care of herself and her property; but if you should come to an opposite conclusion, that she is now a person of *unsound mind*, and incompetent to take care of herself and her property, then, I shall have to point out to you the second question—namely, from what particular period, according to the evidence, you think that she has been in that state. Your duty, therefore, will not be ended by merely stating that she is a person of *sound mind*, or of unsound mind, unless you should come to a conclusion, that having been of unsound mind for a certain period, you think now she has perfectly recovered, and fit to be entrusted with the care of herself and property, or that she enjoys what in law is called a "lucid interval." If, on the other hand, you think she is really a person of unsound mind, you will have to point out, from day to day, the period she has been so. There are three questions before you: Whether this lady is now a person of sound mind, or a lunatic, or a person of unsound mind. If the latter, you must, as I told you, point out the particular period of her having been in that state; unless it should be proved to your satisfaction that she is not a person of unsound mind.

Now, gentlemen, the reason you are called upon to make an inquiry of this kind is, that if this lady should be in that unfortunate state, in which she is alleged to be by the parties who have taken out this Commission, some protection should be thrown about her, and that her property might be taken care of. So that the real object is, that if she should be in that state in which she is alleged to be—namely, in an unsound state of mind, that some friends, or some relation, may be appointed by the Chancellor to be responsible for the safety of her person and her property, and see that she has at command what is necessary for her every essential comfort. You will hear the evidence given, and from the evidence that will be laid before you, you must form your conclusion. You will also see the lady, and examine her yourselves, and this will assist you in coming to a proper conclusion. The medical men in this case will describe the nature of the alleged delusions. I have seen the lady to-day myself, hoping that I might have persuaded her to attend here; what passed is of course not evidence, and therefore it will be needless my repeating it, but I wanted to save you a great deal of trouble. She cannot come here, and some of you will have to go with me in order to see her.

Mr. Lloyd then rose, and stated the facts of the case briefly. After apologising to the jury for the very inadequate manner he felt he should discharge his duty, in consequence of a temporary indisposition, he said his task was very much relieved by the very clear, lucid, and comprehensive manner in which the learned Commissioner had explained the nature of the Commission. The alleged lunatic came over to this country some years ago. In 1815, she married Mr. Theodore Battaglia, who was probably, from the name, an Italian. She lived with him as his wife until the year 1846, when he died; since that period, she had lived as widow on the same premises with a maid servant. She was now upwards of eighty years of age, and it was, therefore, perhaps rare that an inquiry of this kind should be instituted. He was not aware whether any attempt would be made to impugn the motives of the petitioners in this case. If this should be proposed, an opportunity will be afforded him of explanation. Since the death of her husband, she has, as he should show them, exhibited some of the most extraordinary delusions. The learned Commissioner informed them, that there existed no positive definition as to what constituted unsoundness of mind, and that what, in point of fact, may in one person be termed eccentricity may in another be actual insanity. But in the present case he believed there were some characteristic signs of insanity, which would be manifest and clear to all. He had no doubt, himself, that in the present case there was a positive disorder of the understanding; but the hallucinations which existed, would be clearly shown to the jury. He would call a witness who had lived with Mrs. Battaglia from September, 1842, until May, 1847, who would detail her strange extravagance of conduct and passions of extreme violence; her delusions as to infernal spirits. He (the learned counsel) had no doubt but that some attempts would be made to mitigate all these into eccentricities of habit and conduct; but he thought the line between eccentricity and insanity was in this case too clearly marked—a disposition to obscene language would be found in this case, which was quite remarkable in a person, and a lady too, of that advanced age. She has, it seems, imagined that obscene practices took place immediately about her house; she was incoherent that morning when visited.

Perhaps it would be a convenient date, to mark the insanity from Nov., 1846, seeing that she has been of unsound mind since the death of her husband.

Emma Wood was examined by the Commissioner.—Mrs. Battaglia was married in 1815, and Mr. Battaglia died in 1846. Witness then deposed as to her going to take the situation of Mrs. Battaglia, and living with her in the lifetime of her husband. Mrs. B. used to make use of very bad language, though not so bad at the first part of the time that witness lived with her, as at the latter part. In five or six months after witness went to live there, she threatened to cut witness's throat; and about twelve months after, she used to come and sit in the kitchen with me, and frequently threatened to cut my b——y throat. There was also a boy, whose throat she likewise said she would cut. She ran after witness to cut her throat, but witness escaped in the back place, and she called in a great passion, through the door, declaring vengeance against her.

By Mr. Lloyd.—She used to swear she would cut witness's throat, if she did not get out of her place; and the master told witness to take no notice of her, because she was a person of unsound mind, and he engaged witness, therefore she could not discharge witness. It was at the dinner-table that she first threatened to cut witness's throat, when she distinctly said, if witness did not get out of the room, she would cut her b——y throat.

Cross-examined by Mr. Lucina.—Witness was quite positive that it was at the dinner-table, when she made use of this expression.

Mr. Lucina.—Well, now let us hear what she used to say to you?

She used to say I didn't suit her, and if I didn't get out of her way, she would cut my b——y throat. "I will directly, if you don't get out of my house."

Did she say anything else?—No, sir.

You are sure she said nothing else, are you?—She told me to take off that "hell-fire cap" of mine, or else she would tear it off.

When was it she did that?—About a twelvemonth after I went there.

Did she ever say anything to you about devils, or anything of that kind? —Yes, often about devils.

What was it, then?—She sometimes said that devils were in the windows; and that she could hear the burning mountains afar off; but the devils she often saw.

Now, was that more than once?—Several times, sir—she was in the habit of repeating it. There was scarcely a day passed, but that she would repeat those words.

Did you not hear her speak of the noise in the kitchen?—Oh yes, sir.

When?—About three or four years ago.

What was it?—She used to say, "What noises have you got in the kitchen?—what are the serpents about? They are running away. You have got a lot of serpents, I say;" she would bawl out, in a passion—" Get out, the devil and his imps."

Do you recollect the shed being built near the house?—Yes.

How long was that ago?—About three or four years ago. She went round the building, and asked them what they were building, for she supposed they were going to boil her in it when cut up.

By the COMMISSIONER.—Do you mean her or you?—To cut her up, and boil her in, and make soup of.

How did she conduct herself towards her husband?—She treated him in the same way. She has talked of cutting his throat; and witness remembers her jumping up from the dinner-table, and getting a knife and holding it in her hand, and threatening to do it for him.

Cross-examined by Mr. LUCINA.—She came down in the kitchen, and took up the meat spit once, and was going to kill witness with it. She was most violent at times. She frequently thought the devil was dancing and jumping about the place. She often said she was positive she heard voices in her bed-room, when there was nothing of the kind there.

This witness was examined, re-examined, and cross-examined, at very great length, but the substance of her evidence has been given, and it will, therefore, be useless to dwell further upon it; except that the witness added that Mrs. Battaglia was particularly filthy in her habits.

Mr. LUCINA.—Do you mean to swear, or state, or persevere in the statement, that Mrs. Battaglia was so filthy in her person, at the dinner-table, and in the presence of her husband?—She used to make messes in the room.

That you mean to swear?—Yes, sir.

Now, from what time?—It was during the latter part of the time that master was alive.

Dr. ED. J. SEYMOUR was next examined. He had had considerable experience in cases of insanity, both in private practice, and during the many years he had acted as one of the Commissioners in Lunacy. Had written several works on the subject.

Mr. LLOYD.—What is your opinion as to the state of this lady's mind? —I consider her of unsound mind.

Are you able to form any opinion as to whether that unsoundness would be of recent date?—I should think, reasoning from what I have seen of the lady, that it had come on gradually—that is to say, the indications are certainly those of gradual insanity; and gradual unsoundness of mind.

A JUROR.—Have you seen her before?—I have.

The COMMISSIONER.—What questions were put to her in your presence as to the state of her mind?—She was asked whether she had seen Mr. N—— lately and Mr. Jones, whom she had abused in the most violent way.

Well; did she say she had seen them?—Yes; and answered their questions.

Did you examine her as to giving orders to her servants?—No; I consider those questions quite unnecessary.

Then am I to understand that those questions were not put to her?—Something was said to her about money—I cannot remember what it was.

Did she say why she abused the parties you have mentioned?—She did not give any distinct reason; but she said that rascal Collings had been at the window, and she thought he was the very devil himself. When I asked her about Mr. Jones, she said she had not seen him.

COUNSEL.—I believe I understand from you, that in your presence no inquiry was made with respect to her property, or her capability of managing her own ordinary affairs?

The Solicitor.—Yes; but do you mean those questions about pounds, shillings, and pence?—No.

Then did you think those questions necessary, in order that you might form an opinion as to the state of her mind?—Certainly not. She might be quite competent to answer those questions, and yet be of unsound mind.

Now, do you say that she is incompetent to manage her own affairs?—Yes; I never saw a clearer case in my life.

Well, now, supposing that she was unable to recollect the amount of the butcher's bill, would you, on that account, say that she was incompetent to manage her own affairs?—No, not at all.

The Commissioner.—You only saw her once, I believe?—That's all.

How long do you suppose you were then with her?—About an hour.

Did you have the opinion of any other professional man?—It was so conclusive, that I did not require a second opinion.

What age do you suppose her?—About eighty. She looks about that; and, in fact, I was told that was her age. She is most incoherent in her language; sometimes she says one thing, and at another she says exactly the opposite.

You say, when you saw her, she was of unsound mind. Now, do you think she could be imposed on?—Yes; most certainly.

Was she at all energetic in her manner?—She was violent; and particularly when speaking of Lord Brougham, and one or two others.

Mr. Lloyd.—If she had been asked about her property, and the answer was consistent with that which was the fact, would you have altered your impression with regard to the state of her mind?—Not the slightest. Persons labouring under such a state are often exceedingly clever about those things, and yet perfectly incompetent to manage their own affairs.

You say that persons sometimes are enabled to answer questions respecting their property with correctness, and yet be in an absolutely unsound state of mind?—Certainly; I have experienced it in many cases.

The Commissioner.—You say that she is incoherent:—do you bear in mind that she is an Indian?—Yes, perfectly; but I understand that she came to this country some years ago?

Dr. Forbes Winslow, of Sussex House, Hammersmith, was next examined.—Had for many years directed his attention nearly exclusively to the subject of lunacy and the treatment of the insane; was the author of several treatises upon the subject; and has an establishment of his own at Hammersmith for the reception of insane patients.

When did you first see that lady, Dr. Winslow?—On the 3rd of July, 1847, at her own residence.

Was anybody else with you in the room at the time?—Yes; one of her trustees, and a friend.

Did you enter into conversation with her, with the view of ascertaining the state of her mind?—Yes; the examination lasted nearly one hour. I was in close conversation with her nearly the whole of the time.

The Commissioner.—Tell the jury any particulars of the examination.

Dr. Winslow.—With regard to her manner, she appeared extremely excited. She began a very rambling and incoherent conversation about her servant; and I found it quite—thoroughly impossible—to bring her mind to any one single point of conversation. She appeared to be quite incapable of anything like continuity of thought or idea. I asked her various questions, with the view of testing her capacity, and to none of which I could get a precise, satisfactory, definite, or sane answer. She said that a number of whores congregated nightly about the premises, disturbing her peace of mind. She dwelt with great vehemence on this fact, and said that there existed a conspiracy against her life. She declared that a number of persons collected about the premises, for the purpose of concocting plans against her peace of mind, life, and happiness; also that a number of loose women were in the habit of assembling about the place, who were continually prostituting their persons. I asked her if she were under any apprehensions as to these individuals, and she said, "Yes." I asked her twice, and she said she was. She seemed in constant terror, and said that it was a satisfaction for her to think that she was under the protection of government; she also believed that a number of policemen were specially employed to protect her, and that she had only to hold up her finger and she could summon fifty policemen to her aid. The inspector of police, Mr. Mellish, she believed, was especially employed by government to protect her person and property. She then broke out into a rhapsody about Mr. Collings, and said that he came to her in disguise of a tom-cat. She then talked about the great whore of Babylon, and Lord Byron, and quoted texts from the Scriptures, scraps from "Don Juan," and said the whole world was poisoned by whores and papists; subjects that appeared to be exercising a powerful influence on her mind.

But speak generally.—The whole of the conversation was incoherent, violent, and insane.

Did she use coarse expressions?—She spoke without any delicacy of feeling. I have no doubt whatever as to her unsoundness of mind.

And you have seen her since then, have you?—Yes; I saw her again in consultation with Dr. Seymour on the 14th of January. He saw her first, and then I examined her by myself. I found the delusions that I detected on a former occasion still existing. She was quite incapable of holding any reasonable conversation. She still imagined that prostitutes were dancing round the garden.

In your opinion, was she, on either occasions, a person of sound mind, and competent to take care of herself and property?—No; I should say that she was in an unsound state of mind, and wholly incompetent to take care of herself or her property.

Have you any doubt upon the subject?—None.

Having heard the general evidence, what effect has it produced on your mind? Does it confirm or alter your medical opinion?—It very strongly confirms me in my view of the case. I have no doubt the unsoundness of mind has existed for the period that the servant speaks of, supposing her statement to have been true. I should say that this disorder had been coming on for some years.

Do you consider this case a curable one?—I should be disposed to form an unfavourable prognosis. I should say the disordered state of the mind was associated with an organic affection of the brain, which would render the case quite incurable. In giving an opinion of this kind, great caution is necessary. I form my idea of the probable result of the case from the temperament of the patient, the cause of the mental disturbance, the form of the insanity, and the duration of the attack.

Would you have felt any hesitation in certifying her admission into the asylum if it had been desirable?—Not the slightest.

Cross-examined by the Solicitor.—Am I to understand that on neither of these occasions you asked her anything regarding her property?—Her trustee did on the first occasion.

Will you be good enough to tell us what it was she said?—She would not enter upon the question of her property. When the subject was referred to, she broke out into rhapsodies not in the slightest degree connected with the subject. I cannot recollect the precise questions which were asked, but I know that they had reference to some of her title deeds and property.

Did you think it prudent to advise her trustee to speak to her on that subject?—I did not consider it my duty to advise him. I understood he was her trustee. He was a stranger to me.

Did you not go down for the express purpose of seeing whether this lady was competent to take care of herself?—That was the precise object of my visit.

The learned COMMISSIONER summed up, and the jury, without any hesitation, brought in a verdict of unsoundness of mind, dating from the 18th of Nov. 1846.

---

*Commission de Lunatico Inquirendo, opened at the Swan Tavern, Walham Green, Fulham, before* MASTER WINSLOW, *and a Special Jury, to inquire into the state of mind of* CHARLES RICHARD HARRISON, ESQ.

Mr. ALEXANDER, counsel, and a solicitor attended to watch the case on behalf of the petitioners; and Mr. GIFFARD, counsel, and a solicitor appeared on behalf of the alleged lunatic.

Mr. ALEXANDER detailed the facts of the case. He said: Mr. Charles R. Harrison was the son of a very extensive timber-merchant at Hull. He was now forty-eight years of age. In the year 1837 his father died, and left him a considerable fortune. The son afterwards married his present wife, who was his own cousin, and bore the same name. He married her at no great length of time from the decease of his father, and he then set up in business on his own account, that business being totally dependent on his own exertions; but in consequence of his reckless conduct, the business was placed under the charge of those under him at the time. He then lived at Opton Farm, and gave himself up to sports of the field. A considerable change took place in his affairs, and he left York, and took two farms at Guildford, in the county of Surrey. The Opton valuations and these farms amounted to about 5000*l*. At that time he thought that he could dispose of his property to the Hull Dock Company. He mortgaged some of the property to defray his expenses at Guildford; that produced some embarrassment, and he was obliged to obtain some more moneys. He was then with his wife and two children at Surrey; and it was in the autumn of 1845 that the first symptoms of his insanity appeared. In the November of the year he became exceedingly wild, so much so that the medical men who attended him recommended that he should be watched. He was then placed at an asylum upon the certificate of two medical gentlemen, having previously threatened the life of his own wife. He grew much worse, and refused his food. In July, 1846, he returned to his wife; and soon afterwards, his family thought it advisable that he should go down to Leeds to see Dr. Harrison, an old friend. He went, and threatened to destroy one of the surgeons of that part, named Taylor. He was then recommended to be placed at the asylum in York, when he commenced a correspondence of a dreadful nature to his own wife. Some of them showed clear delusions; he spoke of having been dead and come to life again. In other parts he spoke of being a favourite of hell. During the whole of this time he was still indulging in threats against Mr. Taylor and another gentleman, whom he knew very well, and who was a friend to him. He had concealed the carving-knife upon one occasion, when he expected to meet Mr. Graybourn, one of the objects of his revenge, and against whom he seemed to have a most violent antipathy. In the autumn of 1846 he grew rather better. Dr. Harrison intimated to him that the writing of such letters would not have the effect of releasing him from his place of confinement. In November of that year he wrote to Dr. Harrison, and almost contemporaneously with that he sent one of the most horrible communications ever penned to his wife. He actually forged a letter from Dr. Harrison to the persons at the asylum, and effected his escape; but by means of the electric telegraph he was stopped at Derby and brought back. He then made repeated threats against Dr. Harrison, who had also become an object of his antipathy. On the 4th of February of the present year, his wife and Dr. Harrison visited him, and he received her with the utmost indifference. On the 5th of February he escaped from the asylum, and went to Leeds, and there provided himself with two loaded pistols, and went making inquiries for Dr. Harrison, but was unable to find him. He was next removed to Kensington House, and he behaved exceedingly well to Dr. Philp, but to the attendants he used the most offensive and foulest language. He is excessively filthy in his habits. Latterly he has assumed a great deal of vanity. He thinks that he is the finest person in Europe, the most handsome, and that he has the best voice in the country, superior to any one at the opera.

Mr. Graybourn examined.—Brother-in-law to the alleged lunatic. Mr. Harrison was always a very excitable person, but at the time of his taking the farm, he was in his usual state of mind; he was a man of very temperate habits. Saw him in Surrey in early part of December, 1845, and found him in a fearful state of excitement. Stayed with him some time.

Cross-examined by Mr. Giffard.—Had heard him threaten to take Mr. Taylor's life. In the year 1838, he injured the spine of his back by a fall from a horse. Was not aware that his pecuniary prospects were in a bad state after he had taken the farm in 1844; but did not think he was competent to manage so large an estate. At the end of the year 1835, he had mortgaged his property.

Re-examined by the Commissioner.—Had seen great uneasiness and indecision of character about him. Had not thought it necessary to call in medical advice before 1844. Never was under any impression that he was of unsound mind before that period.

Mr. Henry Taylor, Surgeon, examined by Mr. Alexander.—Attended him professionally in 1844; commenced in November.

What was your opinion as to his state of mind?—At the beginning I thought it was hypochondriac, and my suspicions of his insanity were very strong, but I had no direct proof until the 2nd of December of that year; I then thought he was of unsound mind, and recommended that he should be watched.

Why did you recommend that he should be watched?—I was afraid that he would do some mischief either to himself or to some one else. He laboured under a delusion that he had committed some offence, and that he was to be executed for it as soon as he could be caught. He also thought that his wife and family were leagued against him, and that it was their wish to procure his death if they could. He did not state this, but it was implied by his different exclamations. The delusion that he had committed some crime lasted about five days, and he fancied that my father had removed some of the regions of his belly. Upon one occasion he tore a handful of hair from his wife's head, when he thought he was going to be executed, but witness entered the room and stopped him from doing further mischief. He once seized witness by the throat, but was released by the attendants at the asylum. Remembered his refusing to take his food.

Mr. Giffard examined.—Saw him at Moorcraft in January, 1846. Had never performed any operation on him.

Dr. Hobson deposed that Mr. Harrison was, in his opinion, a man of unsound mind. One of his delusions was that he had been in hell, and that the devil had got hold of him. There was a continual hissing in his ears which was altogether unaccountable. At times he would say that his wife was the prettiest woman on earth, and she was a perfect angel, while upon other occasions he would speak in the most disrespectful manner of her, thus clearly showing his insanity. Nothing could exceed his extravagance in her praise sometimes.

THOMAS ELLIS, superintendent of the asylum in which he was confined, and FELIX BROWN, gave corroborative evidence.

Dr. PHILP, of Kensington Asylum, stated that he knew and had attended Mr. Harrison; had heard him use threats against his wife and against Mr. Taylor. He had contemplated suicide, according to his own account. He said he was quite decided on destroying himself, and that it was very necessary that he should do so; he went about the place in search of his pistols, but could not find them. He also said that he had tried to drown himself, but could not effect that in the asylum so well. He also expressed a wish that I should be examined at this inquiry. He has frequently said that if he went to Russia, the emperor of that country would make him a colonel, or distinguish him in some way or other. He was then determined to enter the Russian army. He had seen a balloon, and it had come so near that he could plainly see that his wife was in it. He also spoke to witness of a property of 30,000*l*. a year, which he said his father had left him. He thought he was an exceedingly rich man. He told witness if witness would never see him again, he would give witness a thousand pounds a year, and a variety of other similar evident delusions which witness could hardly remember.

SIR ALEXANDER MORISON.—Had had many years' experience in the management and treatment of insane persons, and was superintendent of Bethnal House Asylum; was perfectly satisfied in his (witness's) own mind that if Mr. Harrison was liberated, he would be dangerous to some persons, and felt thoroughly convinced that he was a person of unsound mind, and having heard the whole of the evidence, there was nothing in it that would in any way alter witness's opinion, but, on the contrary, rather confirm it than otherwise.

By the COMMISSIONER.—Was satisfied upon the first interview that he was of unsound mind. Witness told him that he came there for the purpose of testing his mind; he was quite aware of that fact. Found him on the first occasion labouring under delusions—perhaps not altogether delusions, but most extravagant ideas. He said that no man could practice any trade and be honest, and witness found that he was a man who had run into great extravagances. On questioning him as to the cause of his excitement, he said that the first cause was the refusal of a check at his banker's. The second was the treatment of Dr. Taylor. Was certain he could not take care of himself and his property. The witness offered to read a letter here, when

Mr. GIFFARD objected, and applied to the court to decline the reading of the letter, inasmuch as it could not have the effect of bettering the position of his client.

Mr. ALEXANDER.—After the admission of my learned friend, there can be no objection to read the communication to the jury.

The Court ruled that the communication should not be read.

The alleged lunatic was here introduced to the jury, and questioned by the Commissioner; but his answers were of such a character as clearly to establish the unfortunate state of fatuity under which he laboured.

Verdict—Insanity, dating from November, 1846.

*Another inquiry took place before the same Commissioner, and a Special Jury, at the Windsor Hotel, Southampton, in the County of Hants, to inquire into the state of mind of* MRS. SOPHIA WHEELER, *on Friday, the 1st of June*, 1849.

MR. WISE, of the Western Circuit, appeared as counsel on behalf of the petitioners, and Mr. J. C. SHARP attended to represent the next of kin. Messrs. Rickards and Walker, Solicitors, of Lincoln's Inn Fields, were also present in support of the Commission.

The precept having been read over to the jury, the learned COMMISSIONER opened the case.

Mr. WISE stated the facts of the case.—Mrs. Wheeler formerly resided at Ryde, but lately at No. 6, Clifton Terrace, Southampton. The petitioners were Messrs. Cole and Russell, solicitors, of Ryde. The Commission was issued by these gentlemen, who were creditors of the supposed lunatic, and also of the relatives of the lady. The proceeding arose chiefly from the fact, that this lady having directed a sale of her reversionary interest in some property she had in the funds, thought proper to refuse to ratify it. The refusal was done in a manner most obviously indicative of insanity, which he should show by the evidence he was about to submit to them.

JANE THOMPSON, examined by counsel.—Had lived at Ryde, and knew Mrs. Wheeler very well; had stayed with her for some time. Believed Mrs. Wheeler to be a person of unsound mind. For some time past, while at Ryde, she had shown signs of insanity.

By the COMMISSIONER.—Considered that she had shown signs of a disordered intellect, by her continued suspicion and distrust of everybody about her. Complaining without the slightest reason of being robbed.

How did she say this?—Why, she would say that robbers had broken into the house, whilst nothing of the sort had occurred; nor were there any reasons for her suspecting such a thing.

What else would she say?—She would declare that the boys had got down to her room wherein she lodged, and that they had attempted to injure her health. That they were conspiring against her for that purpose, and to effect which, they intended to put salt in her food.

Did she say this more than once?—Oh, frequently; and she also said that they wanted to put tobacco over her victuals, in order that it might choke her. She said a number of people intended to effect her ruin—she knew it was so, and could not be otherwise. She was sorry that she should be the object of such continued torment.

Would she talk long at a time in that strain?—Oh, yes, for an hour at a time. And her conduct was always very eccentric.

CHAS. STEVENS deposed that he knew Mrs. Wheeler, and in the month of March, 1847, she bought a house of a Mr. Futcher, of Ryde, for 1500*l.* and after having paid 80*l.*, a deposit, she expressed a wish to give it up, not being satisfied with the contract.

And what was the consequence?—Mr. Futcher would not release her except on payment of 200*l.*, besides a forfeit of the deposit money. After this, she entered into an agreement to sell it to Mr. J. N. Weeks, for 200*l.*, the purchase money remaining on mortgage; but, whilst this matter was pending, she resold the property to Futcher, for 1230*l.*, thereby losing 270*l.*, and she subsequently paid Mr. Weeks 100*l.*, to cancel the agreement with him.

Can you give us any other instances?—On the 5th of March last, she placed herself in the High Street of Southampton, holding a parasol over her head, and to which was attached a sheet of music, and to that pinned two checks, drawn in her favour on the Hampshire Banking Company, by Messrs. Cole and Russell.

Was she asked the reason of such extraordinary conduct?—Yes; and her answer was, that she had been completely ruined.

Dr. Brown, of Ryde, examined by counsel.—Do you know Mrs. Wheeler? —I do; and she rented a house of me.

Did you attend her professionally, sir?—No; but her manner was always of a very eccentric character. I did not see her much; but when I did see her, she was always excessively incoherent, and appeared to be suspicious of persons immediately about her, as if she believed they robbed her, and meant besides to do her some bodily harm.

What did she say to make you think this?—Oh, she stated plainly that she was continually being robbed by persons about her, and that she believed the same parties intended to do her some bodily harm.

Do you happen to know whether she was actually robbed, as she said she was?—I believe not—I don't think she had the slightest grounds for thinking so.

What is your opinion as to her state of mind?—I believe her to be utterly incapable of the management of herself, being now of unsound mind. Had visited her at the request of Messrs. Cole and Co., on the 1st of January, 1849, and stated his opinion, at that time, that she was in an unsound state of mind.

Mr. R. S. Fowler, examined by Mr. Wise.—I am a surgeon, at Southampton, and I have known Mrs. Wheeler for twelve years. She had formerly lived at Southampton, when I was her medical adviser. I visited her on the 26th of March, at No. 6, Clifton Terrace, for the purpose of ascertaining the state of her mind, and found her sitting in the parlour. The apartment was comfortably furnished, except that there was no accommodation, whatever, for sitting, except a high music stool. Of this house she was the sole occupant, being without companion or servant.

What did she say to you?—She said, without my putting a single question to her, that she had put a paper in the window, to explain the Scriptures to little children on Sundays; and, amongst other disjointed sentences, she said, "Wicked men look like owls," and "The sun makes them owls." She also said, "The moon is a spirit." I only smiled at hearing all she said, and presently she said that she had something more to tell me; she then went on in a similar strain, saying, "The sun is a spirit;" and upon my asking whether she really believed all she said, she answered most positively in the affirmative, and continued, "All the great men in the Old Testament were great churchmen; I mean Moses, Abraham, and Solomon. The last was greatest, and he had to visit all the churches mentioned in Chronicles and in Deuteronomy. God blessed him with 5000*l.* and a good deal of cattle." Then she would say, "What makes the sea salt? why, it is the fish and oysters in it that make it so salt, I know."

Did you see her after that time?—Yes, I saw her again on the 28th of the same month, and the 29th also; upon which occasions, she expressed herself in an equally incoherent manner.

Did you think her, then, a person of unsound mind?—She exhibited, in my opinion, the most unequivocal symptoms of a disordered intellect; and I can confidently say, that she was, and is, still in an unsound state of mind, and wholly incapable to manage her affairs.

The learned Commissioner.—Then you have no doubt at all about the matter?—None whatever. I am perfectly satisfied about it.

A deputation of the jury then proceeded to visit Mrs. Wheeler, and the

Commissioner's interrogations elicited all the delusions already mentioned; and, on the return of the company to the court, the result of the visit was made known to those of the panel who did not go, and the jury shortly afterwards, and unanimously, returned a verdict of *unsound mind*, dating the insanity from the 1st of June, 1848.

---

### EX PARTE THE COMMISSIONERS IN LUNACY.

(*Bail Court*, *May* 3, 1849.)

Mr. Peacock moved for a writ of *habeas corpus* to bring up the body of a young lady, the daughter of a baronet. It appeared that the young lady had been insane, and had been in confinement at the house of Dr. Sutherland. After being there some time, Dr. Sutherland prevailed on the mother of the young lady to remove her, as she had recovered, and was quite well. The young lady was removed; but it appeared, instead of being taken home, she was taken to the house of a person of the name of Bostock, who had formerly held some office in Hanwell Asylum. She was there kept under some restraint, never being allowed to go out alone, and was not treated as became her station in life. The Commissioners had visited her, and she had told them that she wished to return home; that she wished for society, and to write letters, and communicate with friends. She also stated that Bostock took liberties, and kissed her in the absence of his wife. The Commissioners also examined Bostock, who, after some hesitation, admitted he had kissed the young lady, who was thirty years of age. The commissioners then communicated with the young lady's mother, and in the end the young lady was removed. The Commissioners then wrote to the mother, requiring to know to what place the young lady had been taken; but the mother refused to give them any information, stating, that as her daughter was not insane, she did not know what right the Commissioners had to inquire into her private affairs. The Commissioners therefore asked that this writ might go, in order that the young lady might be asked by the court whether she was willing to remain where she was.

Mr. Justice Coleridge said it was impossible to doubt but that the Commissioners were actuated by the most proper and honourable motives, but he did not think there was sufficient ground for the application. The young lady had been insane, but the Commissioners admitted that she was not so at the present time. Nobody could doubt that she was no longer under the charge of the Lunacy Commissioners. When the mother heard Bostock had behaved ill, she removed her. It was said she had not been taken home, but courts of justice would become a perfect nuisance in the land if they were to interfere in the affairs of domestic life, when it was supposed that a child was not treated with propriety or liberality, and if parents were to be brought into a court of justice and the secret dealings of the family were to be ripped open. If a principle of that kind should be established, nothing would be more mischievous. Another ground was, that the mother had refused to state where the daughter now was. He agreed that that, coupled with other circumstances, might be a ground for a court to interfere, if there had been proof of any ill-treatment, or of her having been improperly put under restraint, but that foundation was wanted, and the mother declined to give the information because, she said, the Commissioners had no authority to ask the question. Upon the whole, without the least hesitation, he thought the rule ought not to be granted; and having so strong an opinion, he thought it right to stop it in the first instance.

Rule refused.

